# Supplementary material for: Space-Time Dependence of Emotions on Twitter after a Natural Disaster
Source: Int J Environ Res Public Health. 2021 May 16;18(10):5292. doi: 10.3390/ijerph18105292 (PMC8157039; doi:10.3390/ijerph18105292)
Supplement: Supplementary file 1 [file ijerph-18-05292-s001.zip › ijerph-1183744-supplementary.pdf]

**Table S1.** Global univariate Moran's I for all exposure variables used in the study. SD=Standard Deviation, CT= Census Tract. Significance values: *** < 0.001, ** < 0.01, * < 0.05, .<0.1.

| Census tract level exposure | Variable | Moran's I | z-value | SD |
|---|---|---|---|---|
| Socio-economic status | Unemployment rate | 0,2451 *** | 18.9995 | 0.0130 |
| Flooding | >50% flooding in CT | 0.5442 *** | 44.3084 | 0.0123 |
| | ≤50% flooding in CT | 0.1993 *** | 16.2114 | 0.0124 |
| | No flooding in CT | 0.6252 *** | 50.2807 | 0.0125 |
| Degree of building destruction | Affected buildings | 0.3385 *** | 27.3170 | 0.0124 |
| | Minor building destruction | 0.4173 *** | 32.8457 | 0.0127 |
| | Major building destruction | 0.2613*** | 22.0999 | 0.0118 |
| | Destroyed building structures | 0.0542*** | 4.4349 | 0.0122 |

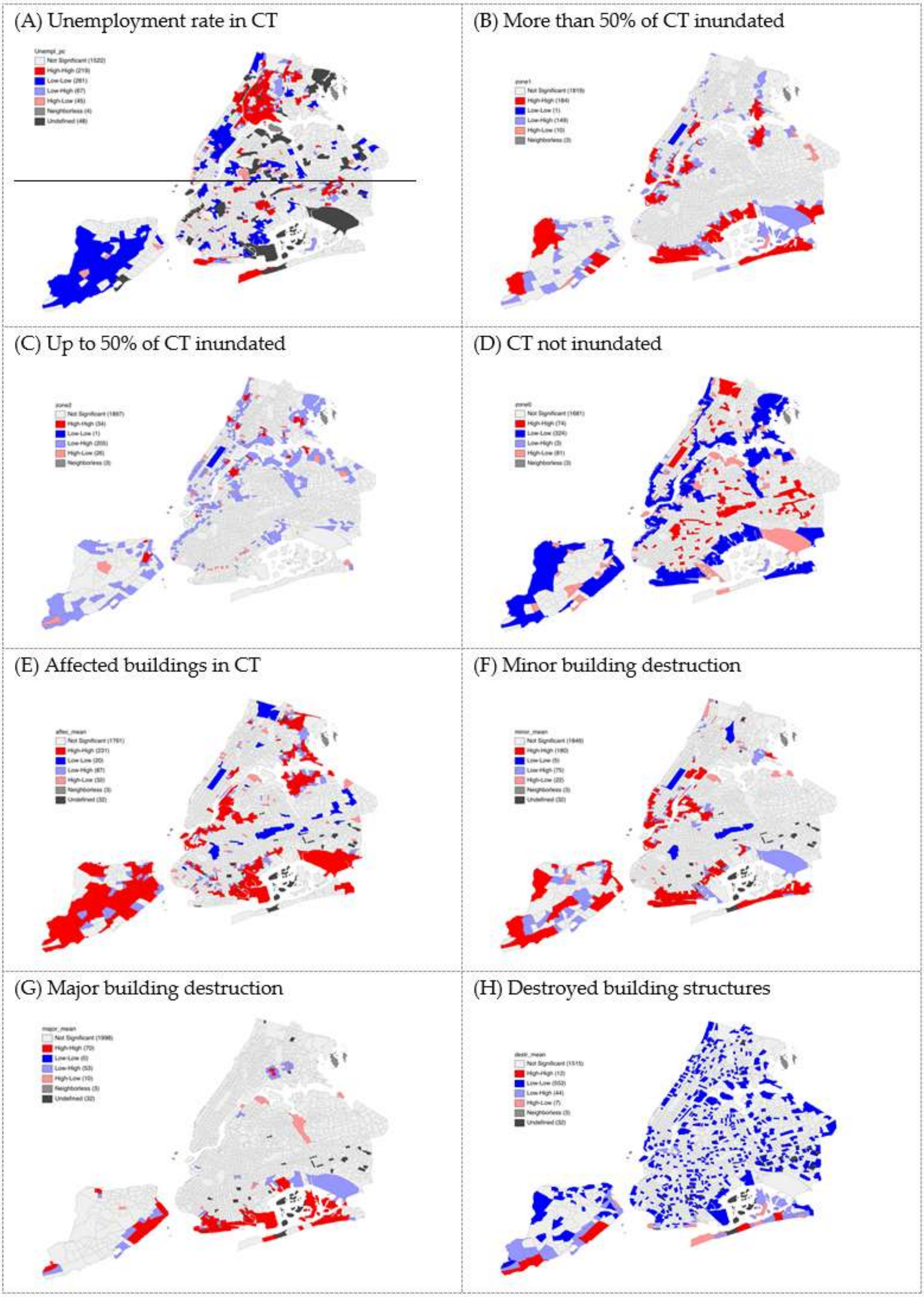


**Figure S1.** Local spatial associations (spatial clusters) of all exposure variables used in the study.
